# The right uncinate fasciculus supports verbal short-term memory in aphasia

**DOI:** 10.1007/s00429-023-02628-9

**Published:** 2023-04-02

**Authors:** Guillem Olivé, Claudia Peñaloza, Lucía Vaquero, Matti Laine, Nadine Martin, Antoni Rodriguez-Fornells

**Affiliations:** 1 Department of Cognition, Development and Educational Psychology, University of Barcelona, Barcelona, Spain; 2 Cognition and Brain Plasticity Group, Bellvitge Biomedical Research Institute (IDIBELL), L’Hospitalet de Llobregat, Barcelona, Spain; 3 Institute of Neurosciences, University of Barcelona, Barcelona, Spain; 4 Legal Medicine, Psychiatry and Pathology Department, Faculty of Medicine, Complutense University of Madrid, Madrid, Spain; 5 Center for Cognitive and Computational Neuroscience, Complutense University of Madrid, Madrid, Spain; 6 Department of Psychology, Åbo Akademi University, Turku, Finland; 7 Department of Communication Sciences and Disorders, Eleanor M. Saffran Center for Cognitive Neuroscience, Temple University, Philadelphia, PA USA; 8 Institució Catalana de Recerca i Estudis Avançats, ICREA, 08010 Barcelona, Spain

**Keywords:** Aphasia, Stroke, Verbal short-term memory, Uncinate fasciculus, Right hemisphere, DTI

## Abstract

Verbal short-term memory (STM) deficits are associated with language processing impairments in people with aphasia. Importantly, the integrity of STM can predict word learning ability and anomia therapy gains in aphasia. While the recruitment of perilesional and contralesional homologous brain regions has been proposed as a possible mechanism for aphasia recovery, little is known about the white-matter pathways that support verbal STM in post-stroke aphasia. Here, we investigated the relationships between the language-related white matter tracts and verbal STM ability in aphasia. Nineteen participants with post-stroke chronic aphasia completed a subset of verbal STM subtests of the TALSA battery including nonword repetition (phonological STM), pointing span (lexical-semantic STM without language output) and repetition span tasks (lexical-semantic STM with language output). Using a manual deterministic tractography approach, we investigated the micro- and macrostructural properties of the structural language network. Next, we assessed the relationships between individually extracted tract values and verbal STM scores. We found significant correlations between volume measures of the right Uncinate Fasciculus and all three verbal STM scores, with the association between the right UF volume and nonword repetition being the strongest one. These findings suggest that the integrity of the right UF is associated with phonological and lexical-semantic verbal STM ability in aphasia and highlight the potential compensatory role of right-sided ventral white matter language tracts in supporting verbal STM after aphasia-inducing left hemisphere insult.

**Supplementary Information:**

The online version contains supplementary material available at 10.1007/s00429-023-02628-9.

## Introduction

The temporary maintenance of different types of information over the time course of their mental processing and representation is essential for multiple cognitive operations. This includes the input and output processing of linguistic information for effective communication. In aphasia, deficits in language processing at different levels of comprehension and production almost invariably coexist with impaired ability to retain linguistic representations in the short term (Martin 2000). Therefore, a comprehensive understanding of verbal short-term memory (STM) deficits in aphasia at both the cognitive and neural levels could provide relevant insights into language-based theoretical models of verbal STM and inform aphasia research and clinical practice. To date, several behavioral studies have helped characterize general STM (see Murray et al. 2018, for a review) and specific verbal STM deficits in people with aphasia (PWA) at the phonological and semantic processing levels (see Martin 2005, for a review). However, only limited research has been conducted to elucidate the brain correlates of verbal STM performance in aphasia. The present study seeks to fill this gap in the literature by characterizing the associations between important white matter tracts and verbal STM performance in aphasia.

Short-term memory (STM) can be thought of as the capacity to store a limited amount of information for a limited time, maintaining it in an active state (Cowan 2008). However, STM is not a unitary maintenance store and can be viewed as part of working memory (WM), a related construct that emerged to account for different types of temporary memory and to incorporate processing in addition to storage operations (Cowan 1996; 2008). The most dominant theoretical model in the field was proposed by Baddeley and Hitch (1974). This multi-component model (Baddeley 2003) entails (i) a limited-capacity central executive control system which seemingly relies on the bilateral frontal cortices (Baddeley & Della Sala 1996); and two storage systems, (ii) the phonological loop associated with left Brodmann areas 6, 40 and 44 (Baddeley 2003) and (iii) the visuospatial sketchpad, which appears to be supported by inferior prefrontal, anterior occipital and posterior parietal regions mainly in the right hemisphere (Gathercole 2008; Papagno 2018). These two storage systems hold verbal and visual-spatial representations, respectively (see Baddeley 2003 for a review). In this influential model, the temporary maintenance of language codes is mainly focused on the storage and processing of phonological information (Gupta and Tisdale 2009). The phonological loop was put forth as a dual-component system with a phonological store that temporarily holds language memory traces, and a process of articulatory or subvocal rehearsal that keeps this information active and accessible. Support for the phonological loop is based on findings from immediate serial recall tasks showing (i) a phonological similarity effect reflected as shorter memory spans when items are phonologically similar (e.g., similar sounding letters and semantically unrelated but rhyming words) relative to sets with phonologically dissimilar items (Baddeley 1966; Conrad and Hull 1964), and (ii) a word-length effect where lists of multisyllabic words are harder to retain compared to single-syllabic word lists (Baddeley et al. 1975). While the phonological loop has been proposed as a “language learning device” that is crucial to facilitate foreign language acquisition through phonological encoding (Baddeley et al. 1998), Baddeley’s model is limited in accounting for the short-term maintenance and processing of semantic information (Baddeley 1966; Cowan 2008).

In the last decades, a growing amount of evidence has pointed towards a further division of verbal STM, with the retention of phonological and lexical-semantic information as two separable components (Martin et al. 1999, 2020; Shivde and Anderson 2011). Dissociations in verbal STM for phonological and lexical-semantic representations have been described across a variety of case studies presenting with selective STM deficits after brain damage. For instance, Martin et al. (1994) demonstrated diverging patterns of verbal STM performance in two patients with acquired brain damage who presented diverging patterns of reduced word spans. Specifically, the first patient presenting a large lesion on the left primary auditory cortex, Wernicke’s area, and the inferior and superior parietal lobules, showed reduced phonological yet normal semantic effects on word spans. In turn, the second patient, who presented with a lesion on the left posterolateral frontal cortex and the left anterior parietal lobule, showed the reverse pattern of memory performance. Moreover, the first patient also exhibited more impairment on a rhyme probe task assessing phonological STM relative to the second patient, who in turn evidenced worse performance on a category probe task tapping lexical-semantic STM. In line with these findings, Majerus et al. (2004) described three patients who had recovered from Landau–Kleffner syndrome, a rare epileptic form of acquired aphasia, but still presented impaired phonological STM on nonword immediate serial recall and rhyme probe tasks, despite normal STM on a lexical-semantic category task. Of note, this dissociation has been corroborated across several studies (see Martin 2005 for a review). All this evidence argues in favor of considering phonological and lexical-semantic STM as distinct capacities that deserve detailed examination, especially in clinical populations with acquired brain damage.

Importantly, the presentation of isolated verbal STM or language deficits alone is rare. Rather, impairments in both domains are generally found together (Koenings et al. 2011; Martin and Saffran 1997; Papagno et al. 2007), in particular when lesions involve brain regions essential for sustaining the interaction and communication between language and memory systems (Roger et al. 2021). Indeed, while verbal STM deficits are uncommon in people with left hemisphere damage without aphasia or with right hemisphere damage (Jodzio and Taraszkiewicz 1999; Kasselimis et al. 2013; Laures-Gore et al. 2011), they frequently coexist with language processing deficits in PWA secondary to brain injury (Martin 2000). There is evidence that phonological and lexical-semantic STM are associated with different aspects of language processing and language learning in this population (see Peñaloza et al. 2022 for a review). For instance, studies on sentence processing in aphasia have shown that phonological STM supports verbatim sentence repetition (Martin et al. 1994; Saffran and Marin 1975), whereas lexical-semantic STM has been associated with the elaboration of phrases during speech production (Martin and He 2004; Martin and Schnur 2019) and the initial retention of word meanings for their integration during verbal comprehension (Martin and He 2004). Likewise, phonological and lexical-semantic STM have been associated with the ability to learn novel word forms and new word-referent mappings in PWA, respectively (Peñaloza et al. 2015, 2016). Moreover, it has been demonstrated that these two STM components make independent contributions to novel word learning in healthy individuals (Peñaloza et al. 2017) and that the functionality of phonological and lexical-semantic learning abilities in PWA can mirror the integrity of their phonological and lexical-semantic STM (Freedman and Martin 2001). In addition, the integrity of verbal STM capacity has been associated with response to language treatment in PWA (Harnish et al. 2018) and interventions aiming to improve verbal STM capacity in this population have shown transfer effects to other linguistic abilities including verbal span and narrative discourse in some cases (Martin et al. 2020). Altogether, this evidence highlights the clinical relevance of the examination of verbal STM in PWA given its potential to inform the diagnosis and characterization of language impairment, and its prognostic value on language treatment outcomes. It also underscores the importance of conducting specific and sensitive assessments that measure verbal STM in terms of the type of linguistic information being encoded, whether lexical-semantic or phonological in nature (Martin et al. 2018), while considering how different language impairment and lesion profiles interact with specific lexical-semantic or phonological STM requirements (Martin and Ayala 2004).

Although the behavioral research mentioned above has helped to characterize verbal STM abilities in aphasia, the number of studies investigating the neural underpinnings of verbal STM is more limited. Both neuroimaging studies (Burzynska et al. 2011; Charlton et al. 2013; Henson et al. 2000; Paulesu et al. 1993; Takeuchi et al. 2011) and lesion studies (Basso et al. 1982; Baldo and Dronkers 2006; Majerus et al. 2004; Meyer et al. 2014; Pisoni et al. 2019; Takayama et al. 2004; Vallar et al. 1990; Warrington et al. 1971) have consistently pointed to the involvement of left-sided brain regions such as the posterior superior temporal gyrus (pSTG) or the supramarginal gyrus (SMG) and frontoparietal tracts, and more specifically the arcuate fasciculus (AF), as supporting phonological STM. On the other hand, the evidence concerning the neural basis of lexical-semantic verbal STM is even more limited. Various fMRI studies involving healthy subjects suggest that the involvement of the left inferior frontal gyrus (IFG) is important for this ability, as measured by tasks such as synonym judgements (Martin et al. 2003; Shivde and Thompson-Schill 2004) or semantic anomaly judgements (Hamilton et al. 2009). Likewise, left IFG lesions appear to be predominantly present in patients presenting with lexical-semantic STM impairments (Hanten and Martin 2000; Martin et al. 1994; Martin and He 2004). In a recent study, Martin et al. (2021) addressed this question by applying multivariate lesion symptom mapping (LSM) in 94 acute left-hemisphere stroke patients. Results for phonological WM as measured with the digit matching span task revealed the involvement of cortical regions such as the SMG, the left inferior frontal junction or the postcentral gyrus—possibly related to subvocal rehearsal as a mechanism to avoid the decay of phonological forms prior to providing a matching response (Baddeley et al. 2021; Chein and Fiez 2001)—as well as subcortical regions including the caudate, the putamen or the lateral prefrontal thalamus. In turn, regions related with lexical-semantic WM as measured by a category probe task included the left IFG, the angular gyrus (AG) and the posterior superior temporal sulcus (pSTS). Although most regions associated with phonological and lexical-semantic WM in the study by Martin et al. (2021) are consistent with previous literature, the proximity—or even partial overlap—of brain regions related to these different verbal STM capacities represent a complicating factor in disentangling their neural underpinnings.

Although maintenance of verbal information appears to be critical for the language system, many models remain vague about the implication and underpinnings of vSTM in language processing. Models focused on language processing (Friederici 2015; Hickok and Poeppel 2007; Jacquemot and Scott 2006) locate verbal STM functions on temporo-parietal areas and their connections with the inferior frontal gyrus. On the other hand, research on verbal STM (Cowan et al. 2011; Martin et al. 1999) proposed that novel phoneme and word serial order might be maintained via a right fronto-parietal network while the maintenance of different verbal stimuli by the direction of attentional control would engage the left fronto-parietal network. Finally, integrative models such as the one proposed by Majerus (2013) advocate for combining the elements of the previous two approaches. Despite the differences in the frameworks presented above, they all seem to converge on the idea that the recruitment of dorsal and ventral language networks is critical for verbal STM, which is possibly tapping on mechanisms for the temporary activation of long-term representations of verbal items to be maintained in the language network. Thus, both dorsal and ventral language streams appear to have a prominent role in verbal STM.

Regarding these language streams, the arcuate fasciculus (AF) has been described as the main white matter pathway supporting the dorsal stream, whereas the inferior fronto-occipital (IFOF), the inferior longitudinal (ILF) and the uncinate (UF) fasciculi are the main white matter tracts related to the ventral stream for language processing (Catani et al. 2005; Dick et al. 2014). Despite the existing evidence supporting the contributions of the abovementioned white matter pathways to phonological and semantic processing, the role of structural connectivity along those tracts in phonological and lexical-semantic STM has not yet been elucidated in aphasia. Considering the high vulnerability of white matter tracts to damage and disconnection following stroke, it is of utmost relevance to assess the white matter structural markers related to the different verbal STM capacities in aphasia.

To this end, the present study aimed to identify the white matter correlates of phonological and lexical-semantic STM in PWA following a left hemisphere stroke. We performed manual deterministic tractography to reconstruct the main language-related white matter tracts bilaterally for each participant and estimated their macro- and microstructural properties by extracting the tract volume and fractional anisotropy (FA) values. All language-related white matter tracts, and especially those with terminations in cortical regions previously associated with verbal STM capacities (Martin et al. 2021) such as the AF, the UF or the IFOF represent good candidates for potentially supporting phonological and lexico-semantic verbal STM in PWA. We further examined the association between these DTI-derived measures and individual scores on phonological and lexical-semantic STM tasks to identify the neural underpinnings of verbal STM in this population, and to gain a better understanding about the white matter tracts that support these abilities after aphasia-inducing brain insults.

## Material and methods

### Participants

Participants were 19 chronic stroke patients (5 females, mean age = 60.5 ± 11.13) who were recruited at three local hospitals: Hospital Universitari de Bellvitge (*n* = 16), Hospital de l’Esperança (*n* = 2), and Hospital Comarcal de l’Alt Penedès (*n* = 1) (Barcelona province, Spain). All participants were diagnosed with aphasia at hospital admission and continued to present persistent aphasia at the time of study enrolment. One participant (P04) who showed scores within the normal limits across different language assessments (described in “Language assessment”) also presented complaints about their everyday language functioning relative to their pre-stroke abilities, indicating that language abilities were not fully recovered. Therefore, the participant was included as impairments in verbal STM measures have been previously reported in people with latent aphasia (Silkes et al. 2021). The following inclusion criteria were employed: (i) age between 25 and 80 years, (ii) Spanish speaker, (iii) right-handed, (iv) unilateral cortical or cortico-subcortical stroke in the left hemisphere confirmed by medical records, (v) at least 6 months post stroke onset, (vi) preserved ability to follow instructions, (vii) eligible for MRI scanning. In addition, none of the participants presented with severe visual or auditory deficits, or a history of psychiatric or neurological disorders other than stroke. Table 1 presents the demographic and clinical information of the stroke participants. All participants provided their written informed consent to undergo study procedures approved by the Institutional Review Board of Hospital Universitari de Bellvitge (reference number: PR224/12) in accordance with the Declaration of Helsinki.

**Table 1:** Demographic and clinical information of the participants

Participants	Sex	Age (years)	Education (years)	TPO (months)	Aphasia type	Aphasia severity rating (1–5)	Aetiology	Lesion location (Left Hemisphere)	Lesion volume (cc)
P01	M	78	4	25	Global	1	Ischemic	Frontal regions, including IFG, MFG and SFG as well as the precentral and postcentral gyri and the rolandic operculum, temporal regions like STG and the ATL, the insula and the putamen	72, 93
P02	M	58	12	40	Transcortical motor	3	Ischemic	Parietal regions (postcentral gyrus and superior and IPL), frontal regions, including IFG, MFG, SFG and the precentral gyrus, the rolandic operculum and the insula	92, 71
P03	M	61	18	26	Anomic	4	Ischemic	Frontal regions, including IFG, MFG and SFG as well as the precentral and postcentral gyri, the STG, the insula and the lentiform nucleus	47, 23
P04	M	62	11	24	Recovered	WNL	Ischemic	The temporal lobe, primarily the MTG and, to a lesser extent, the ITG and STG	9, 20
P05	M	51	5	20	Fluent	3	Ischemic	Mainly the left temporal lobe (STG and MTG) but also parts of the parietal (IPL and precuneus) and occipital lobes	7, 59
P06	F	75	6	20	Fluent	5	Ischemic	The insula, the lentiform nucleus and a portion of the IFG	5, 42
P07	M	63	8	41	Fluent	4	Ischemic	The frontal lobe -primarily the IFG and, to a lesser extent, the MFG- and the Insula	17, 26
P08	F	40	12	14	Broca’s	2	Ischemic	The Parietal lobe (IPL and Postcentral gyrus), Temporal regions (STG and MTG), the insula and the lentiform nucleus	33, 70
P09	M	47	8	6	Broca’s	3	Ischemic	The temporal lobe (STG, MTG, the TTG and the ATL), frontal regions (IFG and the precentral Gyrus) the postcentral gyrus and the insula	31, 29
P10	M	42	14	11	Mixed Nonfluent	1	Ischemic	The entire frontal and parietal lobes, some temporal regions like the STG, the TTG and the ATL, the insula, the lentiform nucleus and the para- and hippocampal regions	186, 40
P11	M	51	13	33	Fluent	5	Ischemic	Mainly the temporal lobe (STG, MTG, ITL and ATL) but also the IFG and the insula	15, 50
P12	M	69	8	24	Fluent	5	Ischemic	Mostly the parietal Lobe (SPL, IPL, postcentral gyrus) and, to a lesser extent, frontal (IFG, MFG, the precentral Gyrus) and temporal (STG, MTG, TTG) regions and the insula	63, 68
P13	M	61	11	34	Fluent	5	Ischemic	The temporal lobe (STG and MTG), the IPL and the insula	5, 63
P14	F	72	6	38	Fluent	5	Ischemic	A part of the lentiform nucleus and the insula	0, 48
P15	F	57	8	9	Nonfluent	3	Ischemic	The frontal lobe (IFG, MFG and precentral gyrus), the postcentral gyrus and the rolandic operculum, the STG and the insula	9, 67
P16	F	71	6	18	Mixed – Nonfluent	2	Undetermined	The frontal lobe (Precentral Gyrus and parts of IFG, MTG and SFG) and also the rolandic operculum, the postcentral gyrus, the IPL, the STG, the lentiform nucleus and the insula	44, 74
P17	M	58	5	36	Broca’s	2	Undetermined	The frontal lobe (precentral gyrus, IFG, MTG), the rolandic operculum, the STG and the insula	23, 18
P18	M	52	11	41	Transcortical motor	3	Ischemic	The frontal lobe (precentral gyrus and parts of the IFG, MTG ad SFG), the rolandic operculum, postcentral gyrus, the STG and the insula	32, 58
P19	M	73	14	31	Fluent	3	Ischemic	Most of the frontal lobe (IFG, MFG, precentral gyrus), parietal regions (IPL and postcentral gyrus), the rolandic operculum, some temporal parts (STG, MTG, ATL), the putamen, the middle occipital lobule, and the insula	77, 89

Demographic and clinical information for each participant. All the lesions described were strictly left-sided

*TPO* time post-stroke, *CC* cubic centimeters, *M* male, *F* female, *WNL* within normal limits, *IFG* inferior frontal gyrus, *MFG* middle frontal gyrus, *SFG* superior frontal gyrus, *ITG* inferior temporal gyrus, *MTG* middle temporal gyrus, *STG* superior temporal gyrus, *TTG* transverse temporal gyrus, *ATL* anterior temporal lobe, *IPL* inferior parietal lobule, *SPL* superior parietal lobule

### Language assessment

The diagnosis of aphasia, the evaluation of aphasia severity, as well as the clinical profile of language and speech abilities of the participants were based on the Spanish adaptation of the Boston Diagnostic Aphasia Examination (BDAE-III) (Goodglass et al. 2005). The assessment of language abilities included the following BDAE-III subtests: (i) naming was assessed with the Boston Naming Test (BNT); (ii) repetition was evaluated with the Sentence repetition subtest; (iii) verbal comprehension was determined with the Word comprehension, Commands and the Complex ideational material subtests; and (iv) reading ability was evaluated using the Basic oral word reading and the Oral reading of sentences with comprehension subtests. Aphasia severity was determined using the BDAE Severity scale and the BDAE Language Competency Index which summarizes each participant’s scores on the main production and comprehension subtests. Finally, verbal comprehension was further assessed with the Token Test (De Renzi and Faglioni 1978) and verbal fluency was evaluated with semantic fluency (animals) and letter fluency tasks (words beginning with the letter P) (Peña-Casanova et al. 2009). Table 2 presents the individual participants’ scores across all language assessments reported in this section.

**Table 2:** General language evaluation scores of the participants

Participant	BDAE-III									Token Test (36 max)	Animal Fluency	Letter Fluency
	Language Competence Index (100 max)	Word Reading (30 max)	Sentence Reading (10 max)	Comprehension in Reading (5 max)	BNT (60 max)	Sentence Repetition (10 max)	Word Comprehension (37 max)	Commands (15 max)	Complex Ideational material (12 max)			
P01	24.16	**6**	NA	NA	**22**	**3**	**26**	**11**	**8**	**14**	**0**	**1**
P02	71.67	30	8	**4**	51	9	37	15	10	**31.5**	**12**	**3**
P03	90	30	10	5	**41**	10	37	15	12	35.5	**13**	**4**
P04	97.5	30	10	5	57	9	37	15	12	34.5	25	9
P05	46.65	30	9	**3**	**42**	9	**29**	**11**	**4**	**28**	**13**	**7**
P06	91.65	30	10	5	50	10	37	15	10	34	10	**4**
P07	79.15	30	10	5	53	9	36.5	15	11	31.5	**12**	**6**
P08	55.8	**23**	**2**	5	**38**	**4**	37	14	10	**20**	**6**	**5**
P09	71.65	30	8	5	48	7	37	15	10	34.5	**13**	**2**
P10	40.83	**20**	**1**	**3**	**41**	**2**	**32**	**11**	10	**12.5**	**4**	**1**
P11	95.85	30	10	5	58	8	37	15	11	35	**16**	**9**
P12	87.5	29	8	**4**	49	9	37	15	10	**28**	22	9
P13	83.33	30	10	5	51	10	36	14	12	32.5	**14**	10
P14	89.16	30	10	5	55	9	35	15	12	32	24	12
P15	60.835	**27**	10	**3**	46	8	**34**	15	**5**	**29.5**	**6**	**3**
P16	47.5	NA	NA	NA	**39**	9	34.5	13	**7**	**19.5**	**5**	**3**
P17	49.16	**27**	7	5	**41**	**4**	**32**	15	**4**	**25.5**	**7**	**4**
P18	64.16	30	10	5	53	9	37	14	**6**	**28**	**13**	**5**
P19	49.16	30	6	**2**	**22**	8	**34**	**10**	**4**	**21**	**4**	**4**

Bold numbers indicate scores that fall below the normal limits according to healthy participant normative data. Scores on the BDAE-III below the 50th percentile according to normative data from PWA considered for the development of this diagnostic battery are marked in bold

*BDAE-III* Boston Diagnostic Aphasia Examination-Third Edition, *BNT* Boston Naming Test, *NA* not available

### Assessment of phonological processing and verbal STM

A selection of subtests from the Temple Assessment of Language and Short-Term Memory in Aphasia (TALSA; Martin et al. 2018) available in Spanish were administered to all participants to evaluate phonological processing and verbal STM, and composite scores were computed as done in previous aphasia studies (Peñaloza et al. 2016, 2017). Table 3 reports the scores of each participant on the described tests.

**Table 3:** Phonological processing and verbal STM composite score for each patient

Participant	Phonological Composite Score	Verbal STM		
		NW Repetition	Pointing Span	Repetition Span
P01	0.6875	0.166	1.5	1.9
P02	0.9875	0.566	3.8	3.8
P03	1	0.6665	4.5	5
P04	1	0.865	5.7	6.2
P05	0.875	0.433	2.9	2.8
P06	1	0.53	3.8	3.6
P07	0.975	0.633	4.8	4.8
P08	0.975	0.3665	2.8	3.3
P09	0.975	0.466	4.2	4.7
P10	0.975	0.2995	2.2	1
P11	1	0.765	4.7	5.1
P12	0.9875	0.233	4.8	4.7
P13	1	0.735	5.4	5.6
P14	0.975	0.8	4.7	5
P15	0.8	0.3995	3.2	3
P16	0.75	0.433	3.2	4
P17	0.925	0.6665	3.1	3.6
P18	1	0.7995	4.4	4.5
P19	0.9375	0.565	3.8	3.3

*verbal STM* verbal Short-Term Memory, *NW* Nonword

#### Phonological processing

Two TALSA subtests were administered to evaluate phonological processing. The rhyming judgments subtest required participants to decide whether pairs of words and nonwords presented auditorily rhymed or not. The phoneme discrimination subtest assessed the ability to discriminate if pairs of words and nonwords presented auditorily were the same or not. Each of these subtests was administered under two conditions with variations in memory load. The 1-s unfilled interval condition presented the words and nonwords of each pair separated by a 1 s delay, whereas the 5-s unfilled interval condition included a 5-s delay between the first and second stimulus of each word and nonword pair. Each condition in the rhyming judgments and the phoneme discrimination subtests included 20 words and 20 nonword pairs. Accuracy across conditions and tasks were summed up into a final phonological processing composite score for each participant.

#### Verbal STM

A set of TALSA subtests including verbal STM measures, either non-lexical (nonword repetition) or lexical (word repetition span, digit repetition span, word pointing span, digit pointing span), were administered to assess different aspects of verbal STM. The nonword repetition subtest assessed the ability to repeat 15 nonwords of 1, 2 or 3 syllables, created by altering one or two phonemes in real words. This subtest included two conditions that required the repetition of nonwords either after a 1-second or a 5-second interval as a way of manipulating STM load. A nonword repetition composite score was calculated by computing the percentage of correct responses in each interval condition and averaging these values across conditions. This composite score represents a measure of *phonological STM with speech output* as stimuli represented phonotactically legal “words” with no lexical-semantic representations. The word and digit repetition span tasks required participants to listen to a sequence of words or digits and repeat them immediately after its presentation, in the same order. The word and digit pointing span tasks required the participants to listen to sequences of words or digits and reproduce them in the same order by pointing at their corresponding pictures on a visual array of 9 possible items (the position of the items within the array was randomized across trials). Each repetition and pointing span task presented 10 strings of stimuli (words or digits) in each of 7 string lengths (1 item, 2 items, 3 items, etc.). In all cases, words and digit names were matched in syllable length, and sequences were generated from a finite set of 9 items, avoiding repetitions within the sequences. The final span size achieved in each task was calculated using the formula: string length at which at least 50% of the strings are recalled + (0.50 × proportion of strings recalled in the next string length), as suggested in previous research (Shelton et al. 1992). The computed spans were then used to calculate two final composite spans: the repetition composite span which averaged the word and digit repetition spans and served as a measure of *lexical-semantic STM with speech output*; and the pointing composite span which averaged the word and digit pointing spans and tapped into *lexical-semantic STM without speech output*. It is worth noting that while the first measure requires the phonological route for repetition and speech output, the second measure can be considered a purer measure of lexical-semantic STM as it does not require speech output (Peñaloza et al. 2016). These three composite verbal STM scores representing *phonological STM with speech output, lexical-semantic STM with and without speech output* were the behavioral variables of interest for this study.

### Neuroimaging data

#### MRI acquisition

All participants were scanned on a Siemens Magnetom 3 T scanner with the Syngo MR B17 software and using a 32-channel head coil at Hospital Clinic, Barcelona (Spain). Diffusion-weighted images (DWI) were acquired with a spin-echo echo-planar imaging (EPI) sequence [TR = 5100 ms; TE = 80 ms; 48 axial slices; 64 directions, GRAPPA (generalized autocalibrating partially parallel acquisitions) acceleration factor 4; slice thickness = 2.5 mm; FOV = 23.5 cm; acquisition matrix = 94 × 94; voxel size = 2.5 mm^3^] with one non-diffusion (b = 0 s/mm^2^) and 64 diffusion weighted volumes (b = 1000 s/mm^2^). A high-resolution T1 (MPRAGE) image was also acquired in the same session (TR = 1970 ms; TE = 2.34 ms; slice thickness = 1.0 mm; acquisition matrix = 256 × 256; voxel size = 1.0 × 0.8 × 0.4 mm).

#### MRI preprocessing

**Fig. 1:**
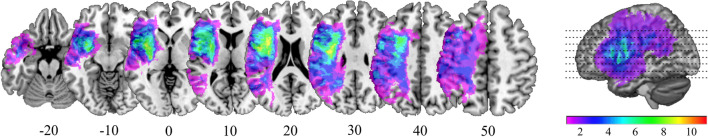
Lesion overlay map. Lesion overlay maps based on lesion masks delineated on T1-weighted images. Montreal Neurological Institute space coordinates of the structural template slices are specified at the bottom of the image and represented by dotted lines on the rendering in the right side of the figure

Prior to preprocessing, all images were visually inspected to ensure the absence of any major artifact that could not be corrected in subsequent steps. Lesions were manually traced slice-by-slice for each participant on their T1 structural brain images by GO using the MRIcron software (http://www.mccauslandcenter.sc.edu/mricro/mricron) and were further verified by an experienced neurologist (see Fig. 1 for the lesion overlay map across participants). Next, as the first step in the preprocessing, T1-weighted images were warped and adjusted to the Montreal Neurological Institute (MNI) space using the Statistical Parameter Mapping software (SPM12, Wellcome Trust Centre for Neuroimaging, London, UK, www.fil.ion.ucl.ac.uk/spm/). The warps obtained were then used to normalize the lesion masks to MNI space. MRIcron was again employed to extract individual total lesion volumes and the xjview toolbox (https://www.alivelearn.net/xjview) was used to identify anatomical structures affected by stroke in each participant (Table 1).

All diffusion images were pre-processed using the FMRIB Software Library (FSL www.fmrib.ox.ac.uk/fsl/fdt) and the Diffusion Toolkit software (DTK) (Wang et al. 2015). DWI data were processed as in previous studies from our team (Olivé et al. 2022; Vaquero et al. 2021) following these steps: (i) eddy-current correction using the FMRIB Diffusion Toolbox (FDT), part of FMRIB Software Library (FSL www.fmrib.ox.ac.uk/fsl/fdt); (ii) brain extraction using FSL Brain Extractor Tool (Smith 2002; Smith et al. 2004; Woolrich et al. 2009) with 0.3 as threshold value; (iii) rotation of the b-vectors; (iv) reconstruction of the diffusion tensors using DTK (Wang et al. 2015); and (v) whole-brain deterministic tractography using DTK with 35 degrees as maximum curvature and a minimum FA threshold of 0.2.

#### Tract dissections

Manual deterministic tractography was performed on preprocessed images focusing on four main language-related white matter tracts: the three segments of the arcuate (AF), inferior fronto-occipital (IFOF), inferior longitudinal (ILF), and uncinate (UF) fasciculi. These tracts were dissected bilaterally for each patient in native space using the Trackvis software (v.0.6.0.1, http://trackvis.org/) by manually placing Regions of Interest (ROI) as described in previous research (Catani and Thiebaut de Schotten 2008; see Olivé et al. 2022 for ROI placement examples of the tracts dissected here).

*AF.* The three segments of the AF were dissected using a three-ROI approach, each drawn in a single slice as described in previous studies (Catani et al. 2005; Lopez-Barroso et al. 2013). The first ROI was delineated in the coronal plane encompassing the fibers going to the IFG, including BA44 and 45 (frontal); the second ROI was drawn in the axial plane covering the white matter fibers traveling to the superior temporal gyrus (temporal); and the third ROI was depicted on the sagittal view, covering the supramarginal and angular gyri (parietal). These ROIs were combined to reconstruct the three subdivisions of the AF: the long (fronto-temporal), the anterior (fronto-parietal), and the posterior (temporo-parietal) segments.

*ILF, UF and IFOF.* To delineate these three white matter pathways supporting the ventral stream for language processing (Hickok and Poeppel 2007; Rauschecker and Scott 2009), we combined three ROIs according to previous studies (Catani & Thiebaut de Schotten 2008). The first ROI was placed axially at the level of the anterior temporal lobe (temporal ROI) encompassing an average of 5 slices; the second one on the anterior floor of the external/extreme capsule covering an average of 3 slices (frontal ROI); and the third one on the region located between the occipital and temporal lobes covering an average of 7 slices (occipital ROI). To define the tracts of interest, we applied a two-ROI approach: the ILF was comprised by fibers going through the temporal and occipital ROIs; fibers going through both temporal and frontal ROIs were part of the UF; and the fibers crossing the frontal and occipital ROIs formed the IFOF.

**Fig. 2:**
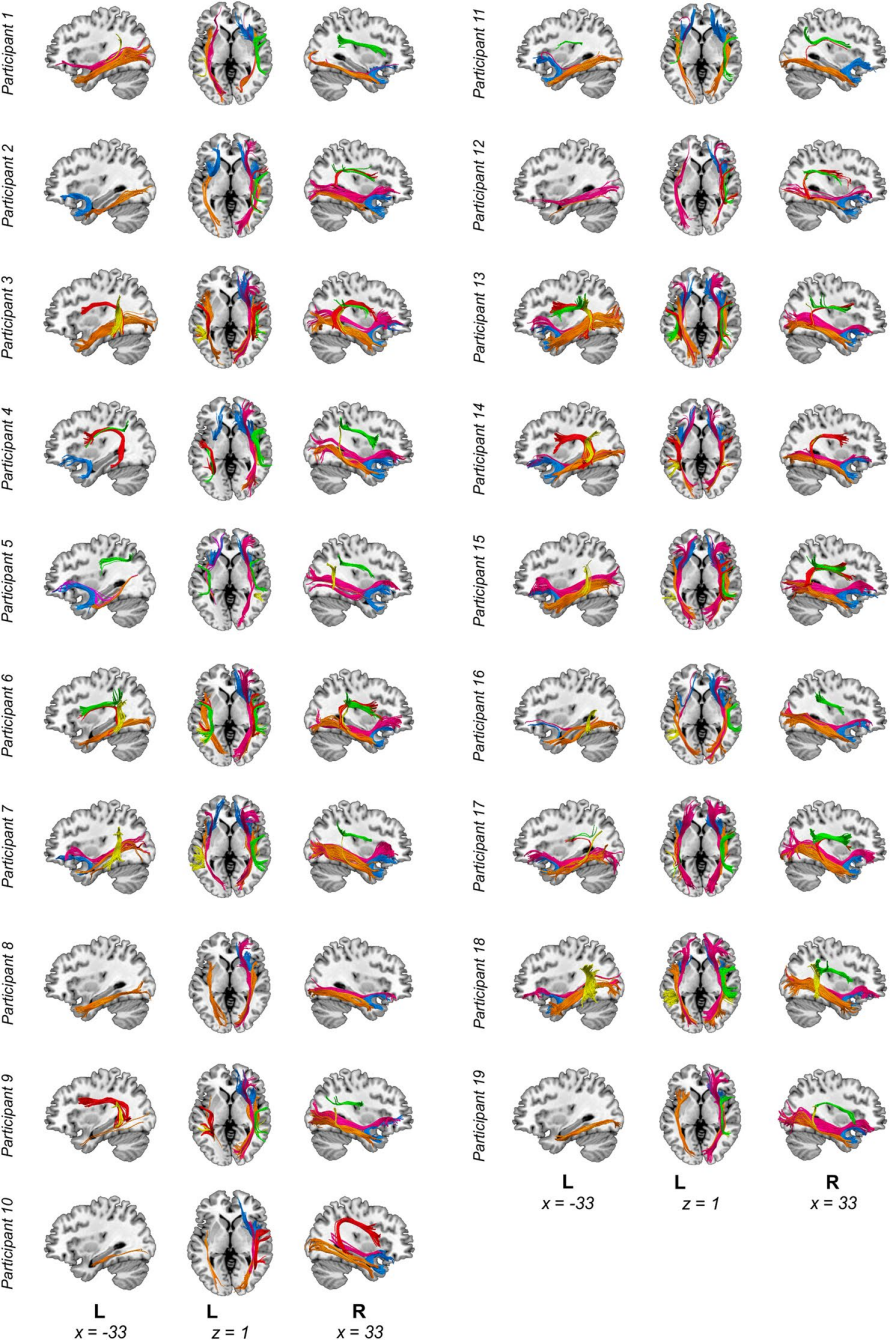
Dissections of all participants. Manual deterministic tractography reconstructions from all participants. Tracts reconstructed were the three segments of the arcuate fasciculus (AF) [Green = anterior, red = long, yellow = posterior segments], Inferior Fronto–Occipital Fasciculus (IFOF) [Magenta], Inferior Longitudinal Fasciculus (ILF) [Orange] and Uncinate Fasciculus (UF) [Light blue]. Abbreviations: L, left. Montreal Neurological Institute space coordinates of the structural template slices are specified at the bottom of the image

Additionally, exclusion ROIs were used for each of the tracts in order to remove any artefactual fibers when present, as commonly done in manual reconstructions (Elmer et al. 2019; Vaquero et al. 2021). For visualization purposes, the streamlines were rendered using the “tube” render option of TrackVis with a radius of 0.15 mm and 10 sides. A depiction of dissections for all participants is provided in Fig. 2.

Output measures extracted from every tract and hemisphere included macrostructural (volume) and microstructural (Fractional Anisotropy, FA) values. Tract volumes are thought to reflect the number of times a streamline could be reconstructed between two brain regions (Jones et al. 2013). Although this measure does not indicate the real fiber count of the tract (Jones et al. 2013), it has been used as a proxy of the tracts’ macrostructure in several DTI studies (Catani et al. 2007; Olivé et al. 2022; Wan et al. 2012) and it is thought to be modulated by properties of the tract including fiber-packing or myelination (Vaquero et al. 2021). As for microstructure, our DTI marker of interest was fractional anisotropy (FA). It reflects the degree of anisotropy (Winston 2012) and numerous intrinsic characteristics including fiber count and dispersion, packing density, myelination or membrane permeability. FA has also been widely used in the DTI literature (Lebel & Beaulieu 2009; Molinuevo et al. 2014) and, together with tract volume, it is considered to be a sensitive measure to explore individual differences (Vaquero et al. 2017). Furthermore, these measures are not only useful for studying healthy anatomy; they also provide valuable information about brain structural connectivity characteristics after a stroke or brain tumor (François et al. 2019; Simó et al. 2015), and have been previously used for investigations in PWA (Forkel & Catani 2018; Ivanova et al. 2016; Schlaug et al. 2009; Yang et al. 2017).

### Statistical analyses

Statistical analyses were conducted using the IBM SPSS software (v25.0). To assess the relationships between white matter macro- and microstructural organization and verbal STM performance in PWA, Pearson correlations were calculated to examine associations between measures of phonological and lexical-semantic STM (nonword repetition, pointing span, and repetition span composite scores) and both mean volume and FA values extracted for each tract and hemisphere. Of note, specific tracts could not be reconstructed for some participants (see Supplementary Table 1 for details on missing tracts per hemisphere). In such cases, volume was computed as zero, whereas FA was removed from the correlation analyses.

The False Discovery Rate (FDR) correction was used to adjust for multiple comparisons and all p values are reported after this correction. FDR corrections were performed separately for each tract and white-matter related measure (6 correlations per tract and measure: 2 hemispheres × 3 verbal STM scores). Additionally, an FDR correction was performed for volume and FA separately with all tracts (36 correlations per measure: 6 tracts/segments × 2 hemispheres × 3 verbal STM scores).

Overall lesion volume was significantly correlated with nonword repetition (r = − 0.498, p = 0.03), repetition span (r = − 0.626, p = 0.004) and pointing span (r = − 0.480, p = 0.038) composite scores. Likewise, aphasia severity (as measured by the BDAE Language Competence Index) was significantly correlated with all three measures: nonword repetition (r = 0.615, p = 0.005), repetition span (r = 0.827, p < 0.001) and pointing span (r = 0.883, p < 0.001) composite scores. Thus, we further examined the contributions of overall lesion volume and aphasia severity to any relationships between white matter measures and verbal STM scores, FDR-corrected significant correlations were reanalyzed as partial correlations using normalized total lesion volume and the BDAE Language Competence Index as control covariates. Of note, the BDAE Language Competence Index was preferred over the traditional BDAE aphasia severity scale for this analysis because it captures a larger individual variability in terms of overall language impairment (range 0–100 percentile scores) while accounting similarly for both comprehension and expression abilities. The BDAE aphasia severity rating scale allows one to classify severity only on a limited 5-point scale which is largely determined by fluency in language production relative to verbal comprehension (Goodglass et al. 2005).

Given the extensive lesions presented by some of the participants, which prevented us from reconstructing some of their left hemisphere tracts, any significant relationship could be influenced by the disconnection caused by the lesion rather than by the overall lesion volume itself. To account for this possibility, we performed a track-wise lesion analysis using Tractotron as implemented in the BCBtoolkit (http://toolkit.bcblab.com/). This method individually compares the lesion mask of every subject to an atlas of the white matter tracts in the healthy adult brain to provide two parameters for each tract: (i) the probability that the lesion intersects a given tract, and (ii) the possible proportion of disconnection of that same tract. Therefore, we extracted these two values for all the left hemisphere tracts and used them as covariates to reanalyze any FDR-corrected significant correlations. On the other hand, other participants (n = 5) presented smaller lesions (< 10 cc) compared to the rest of the sample. To ensure that these less affected individuals did not make an overly large contribution to any possible associations between verbal STM scores and white-matter metrics, all significant FDR-corrected associations were further analyzed excluding these participants.

## Results

### White matter tract volume and verbal STM

**Fig. 3:**
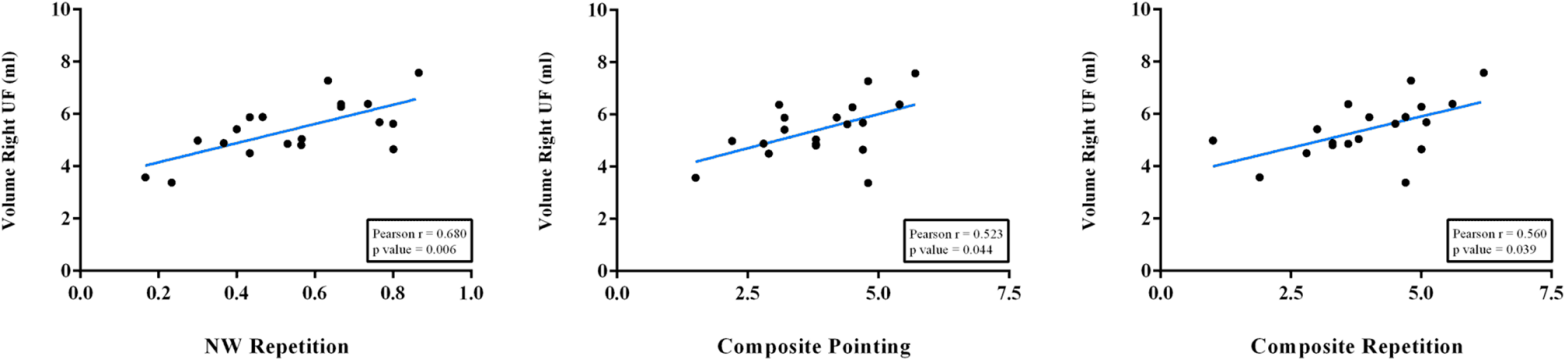
Significant FDR corrected correlation results. Statistically Significant Pearson correlations after FDR correction performed independently for every tract. *P*-values in the figure are already FDR-corrected (6 comparisons)

The right UF emerged as the main white matter tract involved in verbal STM in our cohort of PWA, with tract volume showing significant correlations with all three measures of verbal STM (FDR corrected). Specifically, the right UF volume was significantly correlated with nonword repetition (r = 0.680, p = 0.006), pointing span (r = 0.523, p = 0.044), and repetition span (r = 0.560, p = 0.039) composite scores after the FDR correction was performed independently for every tract (number of comparisons: 6). Figure 3 provides a depiction of these significant associations. Importantly, only the correlation between the right UF volume and nonword repetition scores (r = 0.680, p = 0.036) survived FDR corrections for the multiple comparisons performed for all tracts and hemispheres (number of comparisons: 36). Similarly, partial correlations controlling for both lesion volume and aphasia severity as measured by the BDAE Language Competence Index corroborated this significant association between the right UF volume and nonword repetition scores (r = 0.595, p = 0.012) although its correlations with pointing span (r = 0.426, p = 0.088), and repetition span (r = 0.451, p = 0.069) scores became statistically non-significant.

The results of all the reanalysis using the probability and proportion of tract disconnection as a covariate can be found in Supplementary Table 2. When controlling for the proportion and probability of disconnection of the left UF, the above-mentioned FDR-corrected significant correlations remained significant. These partial correlations also remained significant when using the probability of disconnection of all the left hemisphere tracts dissected as covariates. However, when using the proportion of disconnection of all the dissected left hemisphere tracts as covariates, only the association between the volume of the right UF and nonword repetition remained significant (r = 0.578, p = 0.039), while the associations with repetition (r = 0.477, p = 0.100) and pointing scores (r = 0.407, p = 0.168) became statistically non-significant. Finally, very similar results were obtained when excluding the data of the 5 less affected individuals. Specifically, the analysis with the remaining 14 participants showed a significant correlation between the right UF volume and nonword repetition composite scores (r = 0.712, p = 0.004) while the correlations between the right UF volume and both repetition (r = 0.361, p = 0.204) and pointing (r = 0.388, p = 0.170) composite scores became statistically non-significant.

Additional associations between white matter volume and verbal STM scores were statistically significant, albeit none of them survived FDR correction. Uncorrected significant correlations at the 0.05 level are depicted in Supplementary Fig. 1.

### White matter tract FA values and verbal STM

FA values were not significantly correlated with any of the verbal STM measures for any of the tracts / hemispheres in the present sample (p ≥ 0.05 in all cases). The results from all correlations performed for volume and FA measures are shown in Supplementary Tables 3 and 4, respectively.

## Discussion

The aim of this study was to investigate the white matter structural correlates of phonological and lexical-semantic STM in post-stroke chronic aphasia. Manual deterministic tractography was used to reconstruct the main language-related white matter pathways in the brain including the AF, UF, IFOF, and the ILF. White matter tract volume and FA values were extracted bilaterally for each tract and their relationships with phonological and lexical-semantic STM composite scores were evaluated before and after partialling out the effects of aphasia severity and overall lesion volume. We found that white matter tract volumes, but not FA values, were associated with verbal STM in PWA, suggesting that macro-structural properties of white matter fibers are more sensitive to capture individual differences in verbal STM performance in chronic aphasia. In particular, we found a strong association between the right UF volume and all measures of phonological and lexical-semantic STM. Among these, the strongest association was found between the right UF volume and nonword repetition composite scores after controlling for overall lesion volume, aphasia severity, the disconnection of left hemisphere tracts and the potential contribution of the cases presenting with the smallest lesions in the sample. This result strongly points to a role of the right UF in phonological verbal STM in chronic aphasia.

It is worth considering these findings in light of current neurocognitive models of language processing and verbal STM. Based on the functional specialization of the dorsal and ventral pathways for language processing proposed by these models (Friederici 2015; Hickok and Poeppel 2007; Jacquemot and Scott 2006), one would expect an association between dorsal white matter tracts and nonword repetition composite scores reflecting phonological STM on one hand, and between ventral pathways and repetition and pointing composite spans reflecting lexical-semantic STM on the other. Further, when considering hemispheric lateralization, one would also expect that phonological STM would rely on left lateralized white matter tracts as the dorsal stream for phonological processing is assumed to be strongly left-hemisphere dominant, and that lexical-semantic STM would be supported by ventral tracts in both hemispheres as the ventral stream for semantic processing should be bilaterally organized in neurotypical individuals (Bajada et al. 2015; Hickok & Poeppel 2007). Given these considerations of functional and hemispheric / neuroanatomical specialization, the expectations mentioned above would be particularly relevant to patients examined in the acute/subacute phase after stroke as the functionality of verbal STM (as any other cognitive ability) at this phase would be predominantly reflective of neural integrity (Martin et al. 2021). Nonetheless, our sample exclusively included participants with chronic aphasia, who may have developed specific STM strategies to compensate for their language and verbal STM dysfunction resulting from stroke. Thus, the associations between verbal STM components and the specific white matter tracts and their hemispheric lateralization in this patient sample may reflect some degree of post-stroke functional reorganization. With this consideration in mind, our findings were partially aligned with the above-described expectations in that the volume of the right UF was significantly correlated with both measures of lexical-semantic STM (FDR corrected). This finding supports the classical functional division of the dorsal and ventral streams and suggests that the right UF may still support verbal STM for lexical-semantic representations even after damage to the left UF tract and/or its cortical terminations. This interpretation aligns with the possibility of right hemisphere compensation which may capitalize on the bilateral organization of the ventral stream for semantic processing (Bajada et al. 2015; Hickok and Poeppel 2007).

However, not all correlations between dorsal and ventral white matter tracts and verbal STM measures were in line with the potential associations expected according to models of the dorsal and ventral pathways (Hickok and Poeppel 2007; Dick and Tremblay 2012). Indeed, the volume of the right UF, a ventral white matter pathway, was associated with phonological STM, which would be presumably supported by the dorsal stream. One possible interpretation of these results is that this dorsal-phonological versus ventral-semantic dichotomy may not be as clear as previously proposed, at least in terms of their contributions to different components of verbal STM. Even though phonological processing has repeatedly been associated to the left dorsal stream, some studies have postulated the role of right hemisphere structures, namely frontoparietal tracts, on some aspects of verbal STM such as novel phoneme maintenance and especially word serial order information (Majerus 2013). This would go in line with our results since the strongest association found was precisely between nonword repetition and volume of a right hemisphere structure, in this case the right UF. The invalidation of this clear dorsal-phonological-ventral-semantic dichotomy in relation to the verbal STM would also make sense from an anatomical point of view, given the proximity—or even partial overlap—of the cortical regions that have been previously associated with phonological and lexical-semantic STM (Martin et al. 2021). Moreover, different white matter tracts of either the dorsal or ventral streams of language processing, have terminations in these regions and could constitute structural support for verbal STM abilities. More specifically, the UF is a long-range white matter tract connecting temporal regions including the anterior temporal lobe (ATL), the uncus and entorhinal and perirhinal cortices with the orbitofrontal and lateral prefrontal cortices, the frontal pole and the anterior cingulate gyrus (Dick et al. 2014; Thiebaut de Schotten et al. 2012; Von der Heide et al. 2013). Therefore, the UF presents terminations in inferior frontal regions, which have been associated with both phonological (Chein and Fiez 2001; Yue et al. 2018) and lexical-semantic verbal STM (Lewis-Peacock et al. 2012; Martin et al. 2003; Shvide and Thompson-Schill 2004).

Although its role is still debated (Papagno et al. 2011), the UF is considered as part of the ventral stream of language processing (Hickok and Poeppel 2007), thought to support the mapping of sound-based speech representations to distributed conceptual representations (Saur et al. 2008). Two of the functions most ascribed to this tract are naming and lexical-semantic processing, which have also been attributed to the ATL (Dick and Tremblay 2012; Papagno et al. 2011). Although it has received less attention beyond its role in language, the UF has also been linked to memory functions since it connects the ATL, believed to contribute to semantic memory, and the entorhinal cortex that is related to episodic memory functions carried out in the hippocampus (Von der Heide et al. 2013). Moreover, microstructural properties of the UF have been associated with working memory in normal aging (Charlton et al. 2010) and even to auditory-verbal declarative memory measures in both children (recall measures of word list learning, Mabbott et al. 2009; Schaeffer et al. 2014), and in adults with temporal lobe epilepsy (immediate and delayed auditory memory, Diehl et al. 2008; McDonald et al. 2008), as well as to lexical-semantic learning in healthy young adults (Ripollés et al. 2017). The previously mentioned links between the UF and memory functions support the potential role of this white matter tract in verbal STM. It should be noted that these previous associations have been found between memory functions and white-matter microstructural parameters such as FA, but not with tract volume. However, most of these studies simply did not include tract volume as a variable in their research. Moreover, as previously discussed, FA can reflect various subcellular processes (Winston 2012) and some changes in fiber microstructure may not be reflected in the average FA value even if they have occurred. In addition, the fact that FA is a summary parameter implies that changes in various diffusion directions may remain uncovered (Aung et al. 2013). Thus, the interpretation of the neural correlates of FA values in our study must be done carefully, and it is important to keep in mind that several factors could account for our lack of significant findings concerning the relationship between UF’s microstructure and verbal STM performance.

Notably, while there is a growing number of DTI studies mapping a variety of cognitive functions to specific white matter tracts, the presence of mixed findings and the lower number of studies addressing some white matter tracts relative to others, make it difficult to assign one or more functions to a specific white matter tract. One of the reasons contributing to this difficulty is that the terminations of any given tract can be –and usually are– also connected to other tracts, such that they can form a network of connections with several parallel pathways between two given regions of the brain. The fact that alternative pathways could communicate particular brain regions involved in different aspects of verbal STM (such as the inferior frontal regions) also allows considering that the associations between STM and white matter tracts found in the current study might reflect adaptation processes following stroke. Indeed, brain plasticity mechanisms could account for the possibility that white matter tracts not intrinsically related phonological or lexical-semantic STM could assume these functions following acquired brain injury. For instance, Duffau et al. (2009) argued that the UF is not essential for language, as other tracts of the semantic ventral stream (such as the IFOF) can compensate for it in case of functional alterations. Similarly, previous descriptions have stated that the connection between the posterior superior temporal sulcus (pSTS) and the IFG—at both functional and structural levels—can be supported in alternative ways in addition to the direct physical link provided by the AF (Catani et al. 2005; Friederici 2015), including the UF. This possibility is further supported by studies showing that dorsal and ventral pathways can compensate each other and carry out functions typically ascribed to the other language stream under high demand or functional constraints (Lopez-Barroso et al. 2011; Yeatman et al. 2012) and after brain damage (Rauschecker et al. 2009; Torres-Prioris et al. 2019). In addition, the fact that a right hemisphere tract correlated with phonological STM measures relying on a predominantly left-lateralized dorsal stream, is in line with multiple sources of evidence showing right hemispheric recruitment reflecting compensatory changes in the contralesional hemisphere in PWA following a left hemispheric stroke (see Kiran and Thompson 2019 for a review). In fact, Schneider et al. (2022) recently studied the effect of left-hemispheric stroke lesion location and time post stroke on right hemisphere language activation. Their results revealed that lesions to the left extreme capsule—the anatomical location through which the UF passes through on its fronto-temporal trajectory—are associated with an increased acute to chronic right-hemisphere activation. In turn, the activity of some of these right-hemisphere regions (SMA and IFG) is associated with increased language comprehension performance (Schneider et al. 2022).

To this point, one of the questions that remains open is whether the involvement of the right-hemisphere white matter tracts—especially the UF—in different aspects of verbal STM is intrinsic to these cognitive processes or whether it only occurs as an adaptive strategy to compensate for the lesions observed in the left hemisphere. The premorbid status and volume of right hemisphere tracts might be an important factor defining whether the contralesional hemisphere engages in post-stroke recovery (Kiran and Thompson 2019; Stefaniak et al. 2021). In line with this idea, Forkel et al. (2014) showed that the volume of the right AF was a predictor of the degree of severity of language impairment 6 months after a left hemispheric stroke. As regards to the functional laterality of the UF, the study from Emch et al. (2019) reported a bilateral frontal activation related to verbal WM, which might indicate the involvement of the right UF in healthy individuals. As for its structural lateralization, the previous literature shows inconclusive results regarding the hemispheric differences of the UF (Von der Heide et al. 2013), although some reports point to a right-sided lateralization of the UF when comparing tract volume across hemispheres (Highley et al. 2002). The fact that the UF is not a strongly left-lateralized structure, or that it may even be right lateralized (as opposed to other language-related tracts, such as the long segment of the AF) might somehow facilitate the recruitment of its right hemisphere homologue after a left hemisphere lesion. Nevertheless, although greater right UF volume in healthy subjects might indicate stronger right fronto-temporal connectivity, it does not shed light on whether verbal STM is indeed supported by this structure. Therefore, it is not possible to directly infer its premorbid involvement in verbal STM functions in people with chronic aphasia. While more research is needed to elucidate the role of the right UF in verbal STM in healthy speakers, an asymmetry favoring the right hemisphere suggests that the right UF, as a tract with relatively large volume, could be capable of supporting and assuming cognitive functions such as verbal STM as a result of brain plasticity, especially for PWA with large stroke-induced lesions on the left hemisphere. Another possible interpretation would be that PWA, due to the language processing limitations caused by their brain injuries, may adopt compensatory strategies to complete the verbal STM tasks. In other words, they could rely on relatively more spared phonological mechanisms to perform lexical-semantic verbal STM tasks or vice versa. In fact, it has been previously described that the phonological representation of a word can help reactivate its semantic representation if it is not preserved at the time of evaluation, whereas purely phonological elements might be better remembered if they bear semantic implications (Jones and Macken 2015; Martin et al. 2021). It is important to note that the potential interpretations presented above are not mutually exclusive. Actually, the right UF might support verbal STM in both healthy individuals and in people with post-stroke aphasia, only that in the latter group, this specific support function may especially emerge or increase after brain insult, maximizing the chances to regain verbal STM functionality.

We acknowledge some limitations in the current research, including the restricted sample size which may have reduced the statistical power to identify further relevant associations between white matter tracts and phonological and lexical-semantic STM. This may have influenced the number of significant correlations that finally survived the FDR corrections. In addition, the Language Competence Index was not independent from the verbal STM scores. Likewise, higher lesion volume increases the likelihood that a given tract is damaged. Thus, the partial correlations used may have somewhat underestimated the associations between structural and behavioral variables of interest. Another important limitation is the lack of a control group, which would have helped to clarify the possibility of premorbid involvement of the right UF in verbal STM, given the limited number of studies evaluating the white matter correlates of verbal STM in the healthy adult population. Furthermore, some aspects of the MRI data acquisition and pre-processing steps of the diffusion images could be improved. For instance, future studies could apply a denoising step or the new FSL eddy tool, which should improve to some extent the quality of the preprocessed images and therefore make it easier to detect differences between groups. Unfortunately, the specific imaging acquisition protocol used in this study precluded us from implementing these corrections. Finally, the massive lesions suffered by some of the participants in this study prevented us from reconstructing some of the tracts in the left hemisphere in a notable proportion of the sample. Although this hindered the identification of potential contributions of left hemisphere tracts to verbal STM, our main interest was to identify the white matter tracts that support verbal STM in people with chronic post-stroke aphasia and this constraint is inherent to their condition. Future work should complement our findings by studying white matter tract properties in larger samples of individuals with and without aphasia, in both the acute and chronic states of stroke, and with different lesion extents, in comparison to a healthy control group. This would help to establish if right hemisphere structures intrinsically support verbal STM or to understand if there are tipping points of lesion extent and time post onset that determine the engagement of right tracts over left hemisphere ones. In summary, future research could further corroborate to what extent the associations reported here are reflective of processes of plasticity and reorganization.

## Conclusions

Our findings revealed a strong association between the volume of the right UF and measures of phonological and lexical-semantic STM, with the strongest association being with nonword repetition scores. This suggests that the right UF supports verbal STM in chronic aphasia. These results contribute to a better understanding of the white matter correlates of verbal STM after left hemisphere damage, and cerebral plasticity and compensatory mechanisms in chronic aphasia.

## Supplementary Information

Below is the link to the electronic supplementary material.

Supplementary file1 (TIF 6185 KB)

Supplementary file2 (DOCX 26 KB)

## Data Availability

Anonymized data will be shared by request from any qualified investigator.
