# Supplementary figures and images for: The right uncinate fasciculus supports verbal short-term memory in aphasia
Source: Brain Struct Funct. 2023 Apr 2;228(3-4):875–93. doi: 10.1007/s00429-023-02628-9 (PMC10147778; doi:10.1007/s00429-023-02628-9)

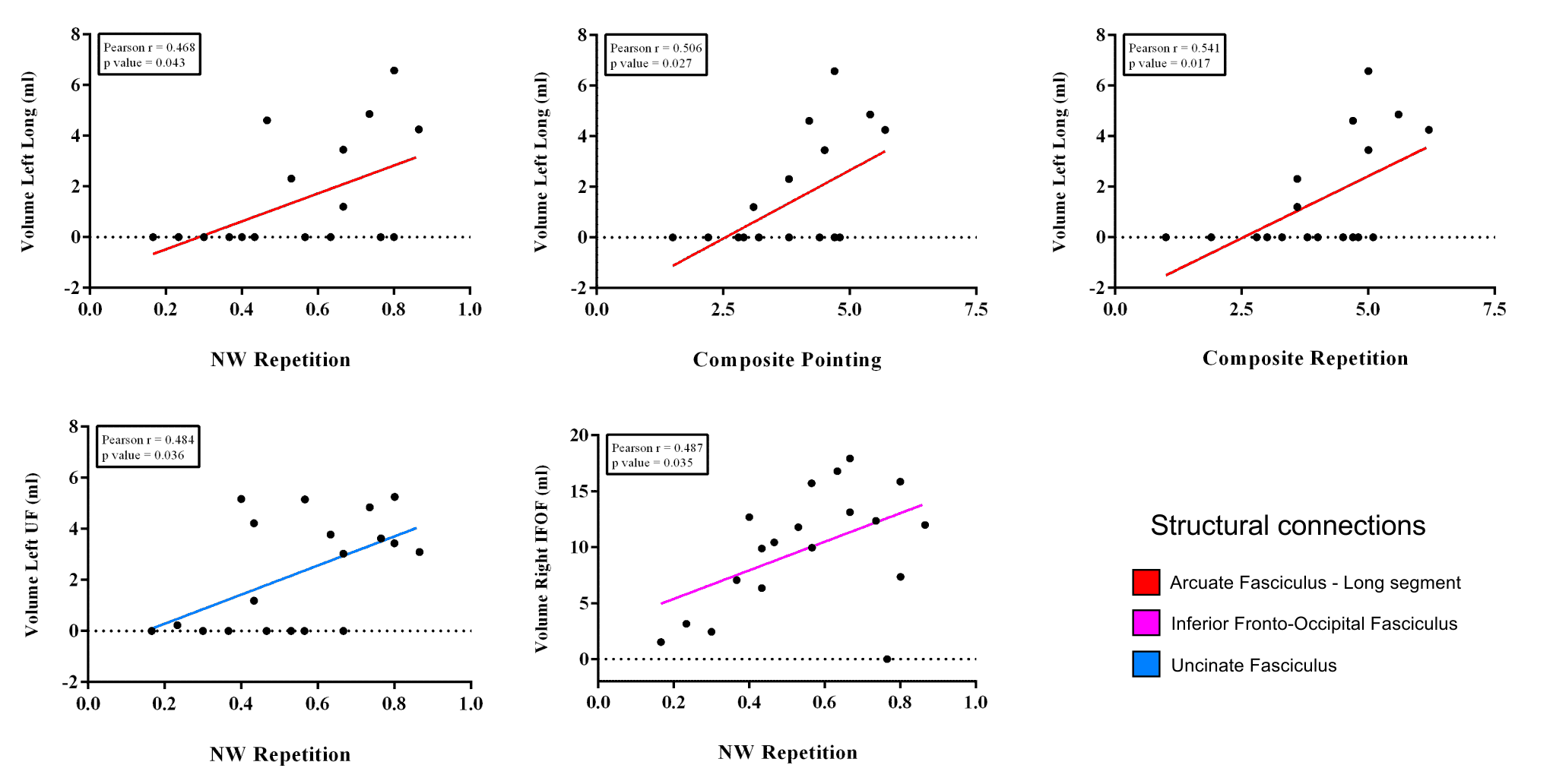

Supplement: Supplementary file 1 — Supplementary file1 (TIF 6185 KB) [file 429_2023_2628_MOESM1_ESM.tif]
