# Supplementary material for: The right uncinate fasciculus supports verbal short-term memory in aphasia
Source: Brain Struct Funct. 2023 Apr 2;228(3-4):875–93. doi: 10.1007/s00429-023-02628-9 (PMC10147778; doi:10.1007/s00429-023-02628-9)
Supplement: Supplementary file 2 — Supplementary file2 (DOCX 26 KB) [file 429_2023_2628_MOESM2_ESM.docx]

**Supplementary Table 1** – Number of missing tracts

| Number of subjects  with **missing tracts** | AF Anterior Segment | AF Long Segment | AF Posterior Segment | IFOF | ILF | UF |
| --- | --- | --- | --- | --- | --- | --- |
| Left Hemisphere | 13 | 12 | 8 | 8 | 1 | 8 |
| Right Hemisphere | 3 | 9 | 7 | 1 | 0 | 0 |

Total number of participants for which each tract or segment could not be reconstructed in each hemisphere with the manual deterministic tractography. Abbreviations: AF = Arcuate Fasciculus, UF = Uncinate Fasciculus, ILF = Inferior Longitudinal Fasciculus, IFOF = Inferior Frontal Occipital Fasciculus.

**Supplementary Table 2** – Details of the partial correlations controlling for disconnection of left hemisphere tracts.

| Covariates | NW repetition x Volume of Right UF | | Pointing Composite x Volume of Right UF | | Repetition Composite x Volume of Right UF | |
| --- | --- | --- | --- | --- | --- | --- |
|  | Pearson r | p value | Pearson r | p value | Pearson r | p value |
| Probability of disconnection of the left UF | **0.749** | **>0.001** | **0.640** | **0.004** | **0.651** | **0.003** |
| Proportion of disconnection of the left UF | **0.669** | **0.002** | **0.501** | **0.034** | **0.574** | **0.019** |
| Probability of disconnection of the left anterior, long and posterior segments of the AF; left IFOF; left ILF and left UF | **0.838** | **>0.001** | **0.646** | **0.017** | **0.703** | **0.007** |
| Proportion of disconnection of the left anterior, long and posterior segments of the AF; left IFOF; left ILF and left UF | **0.578** | **0.039** | 0.407 | 0.168 | 0.477 | 0.100 |

Reanalysis of FDR-corrected significant results as partial correlations controlling for probability and proportion of disconnection of left hemisphere tracts. Bold numbers indicate uncorrected significant results at p≤0.05 level. Abbreviations: UF = Uncinate Fasciculus, AF = Arcuate Fasciculus, ILF = Inferior Longitudinal Fasciculus, IFOF = Inferior Frontal Occipital Fasciculus.

**Supplementary Table 3**– Details of the correlational analysis for all volume measures

| Tract | NW repetition | | Pointing Composite | | Repetition Composite | |
| --- | --- | --- | --- | --- | --- | --- |
|  | Pearson r | p value | Pearson r | p value | Pearson r | p value |
| Left AF Anterior Segment | 0.145 | 0.554 | 0.179 | 0.464 | 0.125 | 0.611 |
| Left AF Long Segment | **0.468** | **0.043** | **0.506** | **0.027** | **0.541** | **0.017** |
| Left AF Posterior Segment | 0.378 | 0.110 | 0.248 | 0.307 | 0.252 | 0.297 |
| Left IFOF | -0.006 | 0.982 | -0.068 | 0.781 | -0.013 | 0.959 |
| Left ILF | 0.366 | 0.123 | 0.138 | 0.573 | 0.177 | 0.468 |
| Left UF | **0.484** | **0.036** | 0.277 | 0.250 | 0.262 | 0.278 |
| Right AF Anterior Segment | 0.357 | 0.134 | 0.164 | 0.503 | 0.219 | 0.368 |
| Right AF Long Segment | -0.110 | 0.654 | -0.068 | 0.783 | -0.216 | 0.375 |
| Right AF Posterior Segment | 0.390 | 0.099 | 0.290 | 0.228 | 0.242 | 0.318 |
| Right IFOF | **0.487** | **0.035** | 0.304 | 0.206 | 0.273 | 0.258 |
| Right ILF | 0.436 | 0.062 | 0.271 | 0.262 | 0.383 | 0.106 |
| **Right UF** | **0.680** | **0.001** | **0.523** | **0.022** | **0.560** | **0.013** |

Uncorrected results obtained for the Pearson correlations performed between the three vSTM measures (NW repetition, Pointing composite and Repetition composite) and volume measures for each tract and hemisphere. Bold numbers indicate uncorrected significant results at p≤0.05 level. Abbreviations: AF = Arcuate Fasciculus, UF = Uncinate Fasciculus, ILF = Inferior Longitudinal Fasciculus, IFOF = Inferior Frontal Occipital Fasciculus.

**Supplementary Table 4** – Details of the correlational analysis for all FA measures

| Tract | NW repetition | | Pointing Composite | | Repetition Composite | |
| --- | --- | --- | --- | --- | --- | --- |
|  | Pearson r | p value | Pearson r | p value | Pearson r | p value |
| Left AF Anterior Segment | 0.054 | 0.890 | 0.102 | 0.794 | -0.052 | 0.894 |
| Left AF Long Segment | 0.381 | 0.277 | 0.546 | 0.103 | 0.465 | 0.175 |
| Left AF Posterior Segment | 0.196 | 0.563 | 0.057 | 0.869 | -0.039 | 0.910 |
| Left IFOF | -0.133 | 0.681 | 0.138 | 0.668 | 0.084 | 0.796 |
| Left ILF | -0.159 | 0.516 | -0.308 | 0.199 | -0.278 | 0.250 |
| Left UF | -0.172 | 0.556 | 0.240 | 0.409 | 0.104 | 0.723 |
| Right AF Anterior Segment | 0.171 | 0.526 | 0.135 | 0.617 | 0.091 | 0.739 |
| Right AF Long Segment | -0.483 | 0.157 | -0.301 | 0.397 | -0.302 | 0.396 |
| Right AF Posterior Segment | -0.352 | 0.262 | -0.109 | 0.737 | -0.094 | 0.771 |
| Right IFOF | 0.124 | 0.624 | 0.353 | 0.150 | 0.419 | 0.083 |
| Right ILF | -0.231 | 0.342 | 0.025 | 0.919 | 0.148 | 0.546 |
| **Right UF** | 0.077 | 0.754 | 0.355 | 0.135 | 0.371 | 0.118 |

Uncorrected results obtained for the Pearson correlations performed between the three vSTM measures (NW repetition, Pointing composite and Repetition composite) and FA measures for each tract and hemisphere. Bold numbers indicate uncorrected significant results at p≤0.05 level. Abbreviations: AF = Arcuate Fasciculus, UF = Uncinate Fasciculus, ILF = Inferior Longitudinal Fasciculus, IFOF = Inferior Frontal Occipital Fasciculus.
